# Transcriptome of Endophyte-Positive and Endophyte-Free Tall Fescue Under Field Stresses

**DOI:** 10.3389/fpls.2022.803400

**Published:** 2022-06-14

**Authors:** Md. Shofiqul Islam, Nick Krom, Taegun Kwon, Guifen Li, Malay C. Saha

**Affiliations:** ^1^Grass Genomics, Noble Research Institute LLC, Ardmore, OK, United States; ^2^Genetics Laboratory, Indiana Crop Improvement Association, Lafayette, IN, United States; ^3^Scientific Computing, Noble Research Institute LLC, Ardmore, OK, United States; ^4^Genomics Core Facility, Noble Research Institute LLC, Ardmore, OK, United States; ^5^Genomics Center, BioDiscovery Institute, University of North Texas, Denton, TX, United States

**Keywords:** abiotic stress, cold, endophyte, RNA-seq, tall fescue, transcriptome

## Abstract

Tall fescue is one of the primary sources of forage for livestock. It grows well in the marginal soils of the temperate zones. It hosts a fungal endophyte (*Epichloë coenophiala*), which helps the plants to tolerate abiotic and biotic stresses. The genomic and transcriptomic resources of tall fescue are very limited, due to a complex genetic background and outbreeding modes of pollination. The aim of this study was to identify differentially expressed genes (DEGs) in two tissues (pseudostem and leaf blade) between novel endophyte positive (E+) and endophyte-free (E−) Texoma MaxQ II tall fescue genotypes. Samples were collected at three diurnal time points: morning (7:40–9:00 am), afternoon (1:15–2:15 pm), and evening (4:45–5:45 pm) in the field environment. By exploring the transcriptional landscape *via* RNA-seq, for the first time, we generated 226,054 and 224,376 transcripts from E+ and E− tall fescue, respectively through *de novo* assembly. The upregulated transcripts were detected fewer than the downregulated ones in both tissues (S: 803 up and 878 down; L: 783 up and 846 down) under the freezing temperatures (−3.0–0.5°C) in the morning. Gene Ontology enrichment analysis identified 3 out of top 10 significant GO terms only in the morning samples. Metabolic pathway and biosynthesis of secondary metabolite genes showed lowest number of DEGs under morning freezing stress and highest number in evening cold condition. The 1,085 DEGs were only expressed under morning stress condition and, more importantly, the eight candidate orthologous genes of rice identified under morning freezing temperatures, including orthologs of rice phytochrome A, phytochrome C, and ethylene receptor genes, might be the possible route underlying cold tolerance in tall fescue.

## Introduction

Tall fescue (*Festuca arundinacea* Schreb.) is a cool-season perennial grass species cultivated in the temperate zone worldwide. It grows well in the transition zone of the United States (Buckner et al., 1979), where cool- and warm-season grasses are cultivated successfully. Tall fescue is highly productive and provides quality forage. It is grown for pasture, hay, and silage and is used as a primary source of herbage protein of livestock feed. Tall fescue can grow in a wide range of temperatures between 4 and 35°C with an optimum 20–25°C for higher biomass production (Rogers and Locke, 2013). It can tolerate cold stress, which includes chilling (0–12°C) and/or freezing (<0°C) temperatures for a short period during fall (Interrante et al., 2020). The forage production is reduced during the cold months, January and February, and growth resumes when temperature rebound to ≥12°C in spring. To maintain reasonable production level throughout the cooler portions of the growing season (Casler and van Santen, 2008), understanding cold stress tolerance mechanism of the cool-season grasses is a prime interest for the grass breeder to create freezing tolerance pasture grass varieties for adaptation under the extreme cold climates.

Freezing tolerance is a complex process that requires cumulative small effect of multiple genes (Zhang, 2009). The major cold signaling pathway is the C-repeat binding factors (CBFs)/dehydration-responsive element binding factor (DREB)-mediated transcriptional cascade, which is essential for the activation of *cold responsive* (*COR*) genes reviewed by Shi et al. (2015). In addition to the *COR* genes, the classical phytohormones, such as auxin, abscisic acid, ethylene, cytokinins, gibberellins, jasmonic acid, and brassinosteroids, are involved in the regulation of plant growth and abiotic stress response (Peleg and Blumwald, 2011). In a non-model plant species, where the genomic sequence information is not available, next-generation sequencing of mRNA (RNA-seq) is a robust method to evaluate transcriptional responses under environmental stress condition. For instances, to improve the understanding of cold/freezing responsive genes, comparisons of transcripts under different temperatures have been conducted in Kentucky bluegrass (*Poa pratensis* L.; Zhang et al., 2016), zoysiagrass (*Zoysia* spp. Willd.), sheepgrass (*Leymus chinensis*; Chen et al., 2013). Transcriptomic analyses have been used to identify differentially expressed genes (DEGs) responsible for the expression of traits within contrasting plant materials.

Tall fescue is a natural host of fungal endophyte (*Epichloë coenophiala*), which produces alkaloids that are harmful to the grazing animals (Waller, 2009), but helps the host to fight against biotic and abiotic stresses (Bacon and Siegel, 1988). To understand the cold stress tolerance in field-grown tall fescue, we analyzed transcripts from the pseudostem (S) and leaf blade (L) tissues of endophyte positive (E+) and endophyte-free (E−) tall fescue genotypes at three different time points in a day from a natural field environment using RNA-seq. Tissue type, cellular conditions, and environmental factors all guided transcript profiles that may influence regulatory events, such as splicing and the expression of genes or their isoforms (Marco-Puche et al., 2019). We thus used two tissue types utilizing E+ and E− tall fescue for transcriptome analysis in this study to monitor the changes in plant gene expressions under cold stress in the natural field environment by considering genetic and environmental interaction, evaluating plant responses, and endophyte’s influence on the host responses. The aims of this study were to: (i) investigate genome-wide transcriptomic profile of E+ and E− Texoma MaxQ II tall fescue, (ii) identify DEGs in two tissues under three time points, and (iii) identify candidate gene(s) responsible for cold tolerance under freezing condition in the natural field environment. Moreover, the present study would provide us useful information whether an endophyte influences host’s genes and their regulatory pathways associated with cold/freezing response in tall fescue.

## Materials and Methods

### Plant Materials

Novel endophyte (AR584) positive (E+) and endophyte-free (E−) tall fescue genotypes of cv. Texoma MaxQ II (referred as “Texoma”; Pennington, United States)^1^ were developed at the Noble Research Institute headquartered in Ardmore, Oklahoma, United States. Texoma is a commercial cultivar freely available for cultivation in the United States. The E+ and E− plants were transplanted in the field for seed production *via* open pollination among them. Since the endophyte does not transmit through pollen, seeds were harvested from the E+ and E− mother plants separately. The seeds obtained from the E+ and E− Texoma genotypes were sown in rows in the experimental farm located at Dupy (Latitude: 34°17′12.106″N, Longitude: 96°59′36.608″W), Gene Autry, Oklahoma. Before collecting samples for transcriptome analyses, we collected S tissues separately in ice-cold 15 ml falcon tubes from 15 each E+ and E− genotypes from three random rows of the plot to perform PCR test to identify their endophyte status, as the endophyte is residing only in the S tissues, not in L (Takach et al., 2012). The S samples were freeze-dried and ground separately in the presence of liquid N_2_ using mortar and pestle. Genomic DNA was extracted using MagAttract 96 DNA Plant Core Kit (QIAGEN Cat. No. 67163, Hilden, Germany) according to the manufacturer’s recommendation. PCR amplifications were performed using primers described in (Charlton et al., 2014) to confirm the E+ and E− status of the Texoma tall fescue genotypes (data not shown).

After confirming E+ and E− status, equal length of S and L tissues were collected on December 10, 2018 under freezing/cold temperatures from the field at: morning between 7:40 am (−3°C) and 9:00 am (0.5°C), afternoon between 1:15 pm (11°C) and 2:15 pm (12°C), and evening between 4:45 pm (12°C) and 5:45 pm (10°C) (Supplementary Figure 1). The 12 samples with three replicates that were collected from the E+ and E− tall fescue at morning, afternoon, and evening were referred as E+MS (endophyte positive, morning, pseudostem), E+ML (endophyte positive, morning, leaf blade), E+NS (endophyte positive, afternoon, pseudostem), E+NL (endophyte positive, afternoon, leaf blade), E+ES (endophyte positive, evening, pseudostem), E+EL (endophyte positive, evening, leaf blade), E−MS (endophyte-free, morning, pseudostem), E−ML (endophyte-free, morning, leaf blade), E−NS (endophyte-free, afternoon, pseudostem), E−NL (endophyte-free, afternoon, leaf blade), E−ES (endophyte-free, evening, pseudostem), and E−EL (endophyte-free, evening, leaf blade; Supplementary Figure 2). In each sample, L/S tissue from 10 genotypes were pooled in a 15 ml tube and immediately frozen in liquid N_2_. After arrival to the laboratory, the samples were stored at −80°C until processing.

### RNA Extraction and Sequencing

The total RNA of each of 36 samples (Supplementary Figure 2) was isolated from approximately 100 mg ground tissues using a Spectrum™ Plant Total RNA Kit (Sigma, Cat. No. STRN250, St. Louis, United States) according to the manufacturer recommendation. The RNA quality was measured in Agilent 2,100 Bioanalyzer (Agilent, Santa Clara, United States) using Agilent RNA 6000 Nano Kit (Agilent, 5,067–1,511), and RNA was quantified using Qubit® RNA BR (Broad-Range) Assay Kit (Life Technologies, Cat. No. Q10211, Carlsbad, United States). The RNA samples were then treated with TURBO DNA-free Kit (Invitrogen, Cat. No. AM1907, Carlsbad, United States) following their protocol.^2^ RNA samples were then cleaned using the RNeasy MinElute Cleanup Kit (Qiagen, Cat. No. 74204, Hilden, Germany) according to the manufacturer protocol.^3^

RNA-seq libraries were prepared using TruSeq Stranded mRNA Sample Preparation Kit (Illumina, Cat. No. 20020594). Briefly, mRNA was purified from one microgram of total RNA, fragmented, and converted to double-stranded DNA for sequencing. Individual libraries were uniquely indexed using TruSeq RNA CD Indexes (Illumina, Cat. No. 20019792), and pooled in equimolar ratio. The pooled libraries were sequenced on an Illumina NovaSeq 6000 150PE Sequencing system.

### Quality Assessment and Assembly of the RNA-Seq Reads

The raw reads of 36 samples (Supplementary Figure 2) were quality trimmed to remove any low-quality bases and primer/adapter sequences before performing the assembly using the Trimmomatic (v. 0.36) using default settings (Bolger et al., 2014). Reads less than 30 bases long after trimming were discarded, along with their mate pair. Endophyte-derived reads were identified by mapping the trimmed reads to the *E. coenophiala* transcriptome (Schardl et al., 2013)^4^ and successfully mapped reads were excluded from further analysis. The trimmed and filtered reads from each sample were independently *de novo* assembled using the software Trinity (v. 2.8.5) with default parameters (Grabherr et al., 2011). These assemblies were then combined by randomly selecting one as a starting transcriptome and then iteratively aligning the transcriptome with each assembly, identifying that assembly’s novel transcripts, and adding those transcripts to the combined transcriptome. Each sample was then mapped to the combined transcriptome using HISAT2 (v. 2.0.5)^5^ with 24 threads and the default mapping parameters (Kim et al., 2019). The expressed transcripts in each sample were quantified using the StringTie (v. 1.2.4) with the default assembly parameters to produce more complete and accurate reconstructions of transcripts and better estimates of their expression levels (Pertea et al., 2015).

### Identification of Differentially Expressed Genes

To identify genes which expressed under different temperature condition during morning, afternoon, and evening time with or without the presence of endophyte, pairwise differential gene expression testing was performed using DESeq2 with default parameters setting (Love et al., 2014). DESeq2 method was used for differential read counts per gene in RNA-seq, using shrinkage estimation for dispersions and fold changes (FC) to improve stability of estimates across experimental conditions. A log2 FC ≤ -5 and ≥ 5 and adjusted value of *p* ≤0.05 were used to determine the significant differences in differential gene expression between two samples. The DEGs with log2 FC with “–” and “+” sign indicates downregulated and upregulated genes, respectively.

### Hierarchical Clustering and Visualization of Differentially Expressed Genes

Hierarchical clustering analysis of DEGs from the six comparisons, E+MS vs. E−MS, E+ML vs. E−ML, E+NS vs. E−NS, E+NL vs. E-NL, E+ES vs. E−ES, and E+EL vs. E−EL, was constructed using the function heatmap.2 in the R package gplots (Warnes et al., 2015) in R Studio. The DEGs that were biologically significant were visualized using the web-based software DiVenn (Sun et al., 2019). The red and blue nodes represent up- and downregulated genes, respectively. The yellow nodes represent upregulated in one dataset but downregulated in the other dataset.

### Identification of Orthologous Genes Using Tall Fescue Transcripts

As annotation of tall fescue genome is not available till to October 25, 2021, the complete and accurate tall fescue transcripts were aligned against the switchgrass non-redundant protein sequences in Phytozome v13 database^6^ using BLASTX searches to identify best-matched switchgrass orthologues. Using switchgrass orthologues, we also obtained rice and *Arabidopsis* orthologues of tall fescue transcripts from the Phytozome database.

### Gene Ontology Analysis of Differentially Expressed Genes

We performed GO enrichment analysis using orthologue genes of rice to identify their involvement in biological process (BP), molecular function (MF), and cellular component (CC) categories. To study the influence of endophyte, the DEGs between E+MS vs. E−MS, E+ML vs. E−ML, E+NS vs. E−NS, E+NL vs. E−NL, E+ES vs. E−ES, and E+EL vs. E−EL were used for GO enrichment analysis. The rice orthologues of the tall fescue DEGs were used as input data to perform GO analysis using singular enrichment analysis (SEA) tool against *Oryza sativa japonica* annotation of the web-based AgriGO v2.0 (Tian et al., 2017) with modified statistical parameter settings: statistical test method, Fisher; multi_test adjustment method, Yekutieli (FDR under dependency); significance level, 0.01; and minimum number of mapping entries,10. MSU7.0 gene ID (TIGR) of rice orthologues was used as reference during SEA analysis.

### KEGG Pathway Enrichment Analysis

The KEGG pathway enrichment analysis was performed using KOBAS 3.0 (Xie et al., 2011) on the basis of Fisher’s exact test with Benjamini and Hochberg (1995) FDR-corrected value of *p* <0.05. The top most significant pathways based on FDR-corrected *p*-values in all six comparisons, E+MS vs. E−MS, E+ML vs. E−ML, E+NS vs. E−NS, E+NL vs. E−NL, E+ES vs. E−ES, and E+EL vs. E−EL were presented.

### Prediction of Candidate Genes Responsible for Cold Tolerance in Tall Fescue

To identify candidate genes associated with cold tolerance in tall fescue, we searched genes of each GO term whose function related to stress, abiotic stimulus, ethylene stimulus, DNA damage stimulus, light stimulus, and radiation etc.

### Validation of DEGs Using Quantitative Real-Time Reverse Transcription-PCR

Eight DEGs obtained from E+MS vs. E−MS were selected for validation using quantitative real-time reverse transcription-PCR (qRT-PCR) analysis. Total RNA treated with TURBO DNA-free Kit (Invitrogen, Cat. No. AM1907, Carlsbad, United States) was used to synthesize the first-strand cDNA using SuperScript III Reverse Transcriptase (RT) kit (Invitrogen, Carlsbad, CA, United States, Cat. no.: 18080044) following the manufacturer protocol.^7^ Briefly, for each RNA sample, the following components were combined in a PCR tube on ice to a volume of 12 μl containing 5 μl DNase-free RNA (200 ng/μL), 1 μl 50 mM oligo (dT)_20_, 1 μl 10 mM dNTPs and 5 μl RNase/DNase-free water. The reaction mixtures were incubated at 65°C for 5 min and then placed on ice for 2 min. The first-strand cDNA synthesis master mix was prepared on ice by adding 4 μl 5x First-Strand buffer, 1 μl 0.1 M DTT, 1 μl RNaseOUT Recombinant RNase Inhibitor (40 units/μL), and 1 μl SuperScript III RT (200 units/μL). The first-strand cDNA synthesis master mix was mixed properly by gentle vortex and was added into the pre-incubated RNA and oligo tube. The reaction mixture was mixed by pipetting up and down and incubated at 50°C for 60 min. The reaction was terminated at 70°C for 15 min and cooled on ice. The cDNA was stored at −20°C.

qRT-PCR reactions were prepared in an optical 384-well plate in a volume of 10 μl containing 2 μl of the forward and reverse primer (1 μM/μL), 5 μl of 2x Sigma KiCqStart SYBR Green qPCR Ready-Mix (Cat no.: KCQS01), 1 μl molecular biology grade water, and 2 μl cDNA (1:20). qRT-PCR amplifications were performed on QuantStudio 7 Flex Real-Time PCR system (Thermo Fisher Scientific, Singapore) using a protocol of 2-step PCR cycle (an initial denaturation of cDNA at 95°C for 3 min, followed by 40 cycles of denaturation at 95°C for 15 s and annealing at 60°C for 45 s) and a 3-step of melting curve analysis (95°C for 15 s, 60°C for 1 min and 95°C for 15 s). Experiments were performed with three technical replicates of each S tissues of E+ and E- tall fescue collected under freezing temperature in the morning. Gene expression was quantified using the 2^-ΔΔCT^ method (Livak and Schmittgen, 2001). The tall fescue *Actin* gene was used as an internal reference gene. Primers used to amplify 53–63 bp of the genes were designed using Primer Express software (v3.0.1; Thermo Fisher Scientific; Supplementary Table 1) and synthesized by Sigma-Aldrich, MO, United States.

## Results

### Sequencing and *de novo* Assembly of the Texoma Tall Fescue Transcriptome

After the quality assessment and data filtered, a total of 553.8 million high-quality paired-end reads were identified in the E+ (18) samples and 484.8 million in the E− (18) samples (Table 1). The filtered reads were *de novo* assembled into 5,520,386 and 5,133,272 contigs in the E+ and E− samples, respectively for downstream analysis. From these contigs, we identified unique transcripts varied from 186,653 to 200,380 in the E+ samples and from 188,468 to 194,606 in the E− samples. Overall, a total of 226,054 transcripts were identified in the E+ samples and 224,376 transcripts in the E− samples. The length of these transcripts varied from 177 to 27,968 bp and the N50 varied from 1,288-1,326. Finally, we found 234,883 transcripts from all the samples collected in this study (Table 1). In addition, the result showed about 5.18 and 0.95% more transcripts were expressed in endophyte-infected S and L, respectively over endophyte-free S and L under freezing temperatures in the morning.

**Table 1:** Assembly and annotation of the transcriptome data obtained from short read Illumina sequencing of Texoma endophyte positive (E+) and endophyte negative (E−) tissues under cold stress in the natural field environment.

Parameters		Texoma E+			Texoma E−			Combined
	Time	Pseudostem	Leaf blade	Pooled	Pseudostem	Leaf blade	Pooled	
Filtered paired-end reads^*^	M	137,172,033	62,692,906	553,802,405	71,139,077	78,140,815	484,814,092	
	N	87,040,508	71,783,216		92,460,683	73,609,080		
	E	86,632,321	108,481,421		95,417,751	74,046,686		
No of contigs after Trinity assembly^*^	M	1,125,143	780,760	5,520,386	817,647	841,846	5,133,272	
	N	929,259	769,068		906,092	799,442		
	E	941,726	974,430		930,388	837,857		
No of unique transcript	M	200,380	190,568	226,054	190,514	188,760	224,376	234,883
	N	193,981	186,653		193,370	188,468		
	E	194,588	193,295		194,606	190,710		
Transcript length (bp)	M	177–16,616	179–15,501	177–16,616	177–16,644	178–15,236	177–27,968	177–27,968
	N	178–15,651	177–15,604		177–15,596	178–16,026		
	E	179–15,718	178–15,966		177–15,590	179–27,968		
N50	M	1,316	1,288	1,288–1,338	1,317	1,303	1,300-1,326	1,288–1,326
	N	1,318	1,338		1,318	1,326		
	E	1,317	1,319		1,300	1,304		

*M, morning; N, noon; and E, evening*.

* *Data presented as sum of three biological replicates*.

### Identification of Orthologue Genes From Other Plant Species

Among the combined transcripts (234,883), about 13.5% got hit to switchgrass (31,780), rice (31,622), and *Arabidopsis* (31,604) genomes. A total of 5,919, 4,476, and 4,002 orthologue genes were identified in switchgrass, rice, and *Arabidopsis*, respectively (Supplementary Figure 3). Due to lack of well-annotated tall fescue genome, the function of the majority fescue transcripts remains unknown when compared to the reference genomes of the related species.

### Analysis of DEGs Between E+ and E− Texoma Tall Fescue in Different Time Points

**Figure 1:**
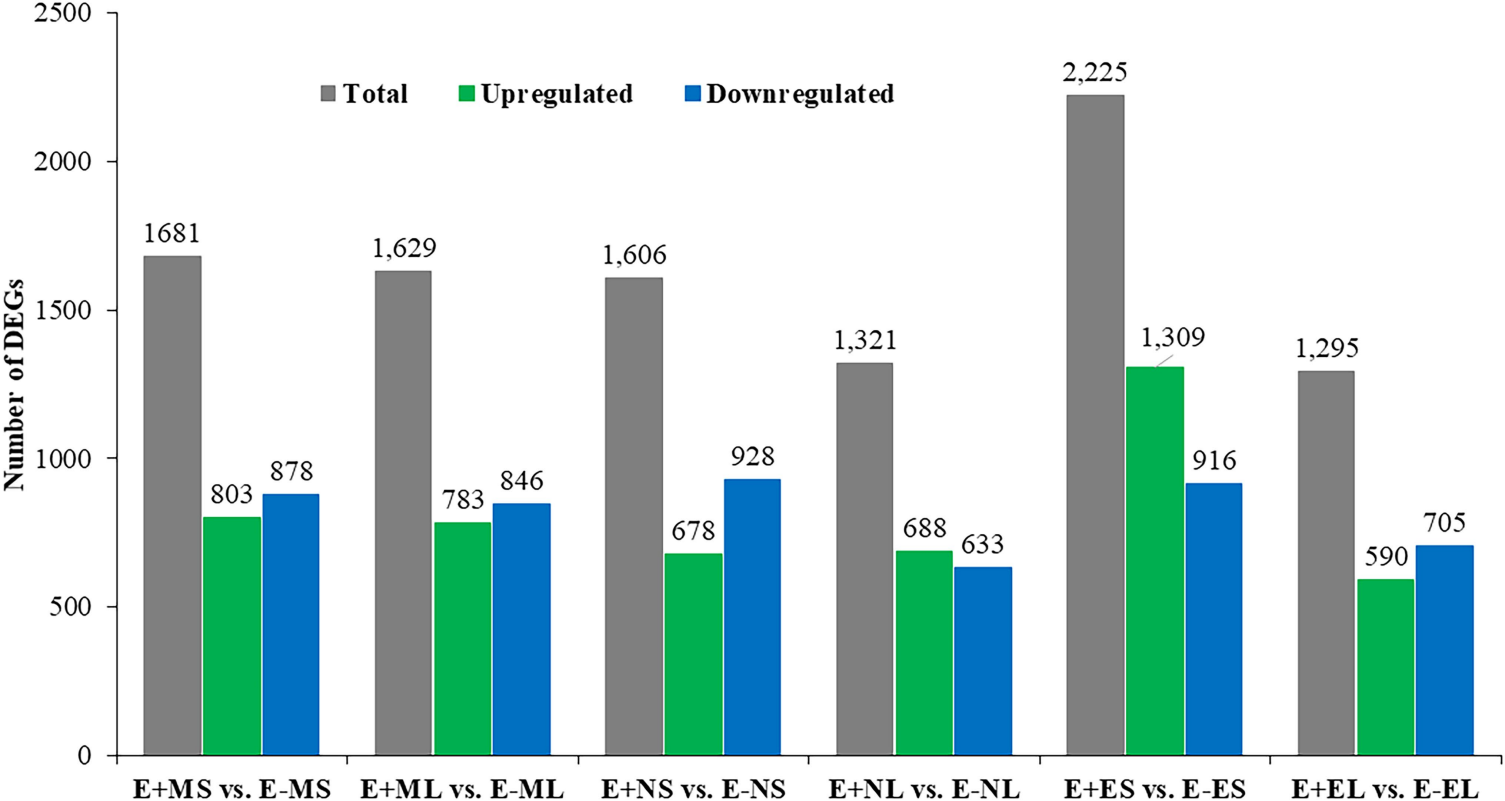
Identification of differentially expressed genes between E+ and E− tissues under cold stress. Samples were: E+MS (endophyte positive, morning, pseudostem), E+ML (endophyte positive, morning, leaf blade), E+NS (endophyte positive, afternoon, pseudostem), E + NL (endophyte positive, afternoon, leaf blade), E+ES (endophyte positive, evening, pseudostem), E+EL (endophyte positive, evening, leaf blade), E−MS (endophyte-free, morning, pseudostem), E−ML (endophyte-free, morning, leaf blade), E−NS (endophyte-free, afternoon, pseudostem), E−NL (endophyte-free, afternoon, leaf blade), E−ES (endophyte-free, evening, pseudostem), and E−EL (endophyte-free, evening, leaf blade).

Differential gene expression in E+ tall fescue was analyzed relative to E− tissues under three different time points under natural field environment. In total, 5,757 significant DEGs (value of *p* ≤0.05) with log2 FC ≤ -5 and ≥ 5 were identified at least in one of the six following comparisons: E+MS vs. E−MS, E+ML vs. E−ML, E+NS vs. E−NS, E+NL vs. E−NL E+ES vs. E−ES, and E+EL vs. E−EL (Figure 1; Supplementary Table 2). Analysis of DEGs showed that higher number of transcripts were expressed in S than L tissues at each time point (Figure 1). The upregulated transcripts were detected fewer than the downregulated ones in both tissues under the freezing (−3–0.5°C) temperature (S: 803 up and 878 down; L: 783 up and 846 down) in the morning. At afternoon (11–12°C), fewer upregulated (678) than downregulated (928) transcripts were identified in S tissues, but it was differing in L tissue (688 up- vs. 633 downregulated). In contrast to the afternoon, in the evening (12–10°C), more upregulated (1,309) than downregulated (916) transcripts were identified in S tissues, and fewer upregulated (590) than downregulated (705) transcripts were detected in L tissues (Figure 1).

**Figure 2:**
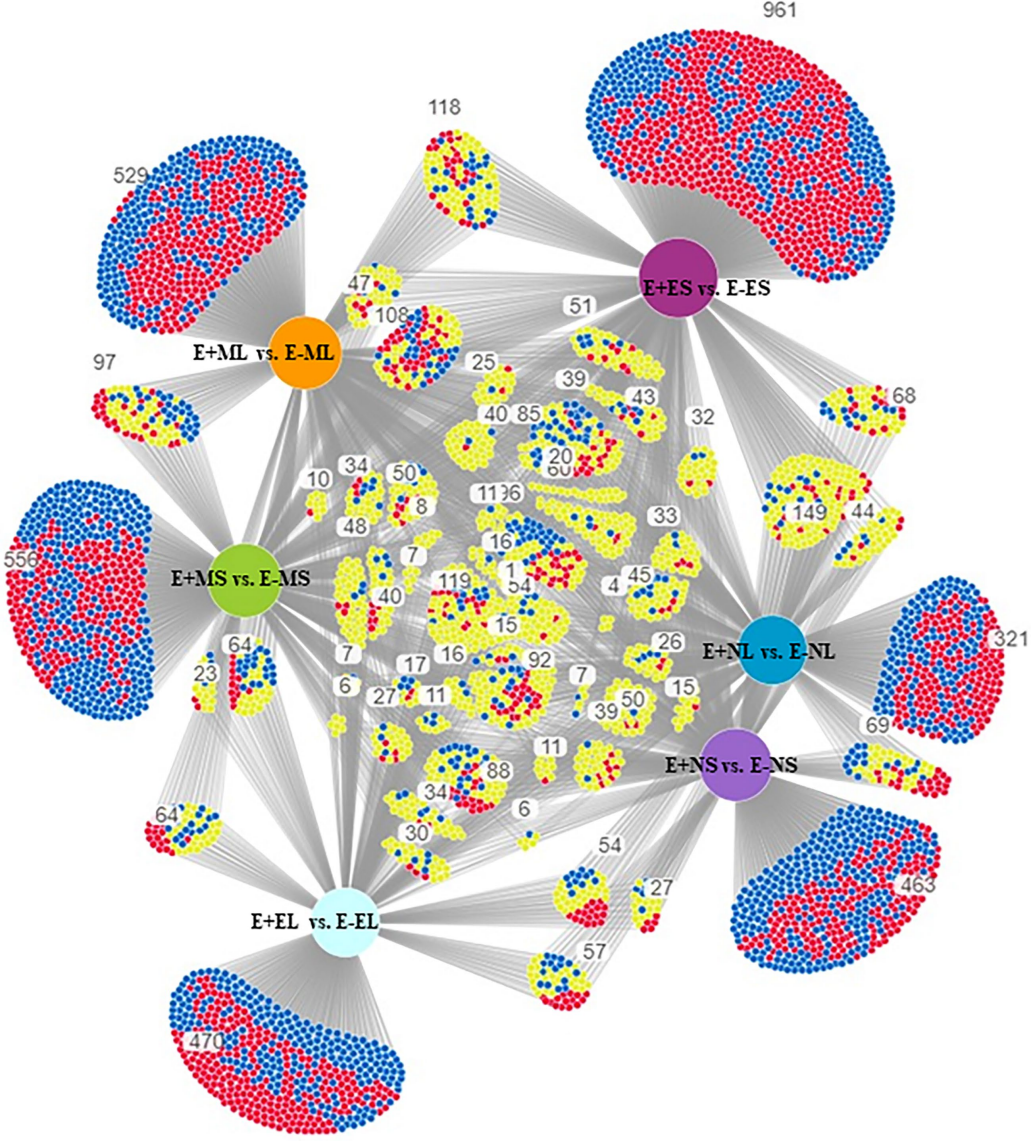
Visualization of differential gene expression pattern among E+MS vs. E−MS, E+ML vs. E−ML, E+NS vs. E−NS, E+NL vs. E−NL, E+ES vs. E−ES, and E+EL vs. E−EL using DiVenn program. The red and blue nodes represent up- and downregulated genes, respectively. The yellow node represents upregulated in one dataset but downregulated in the other dataset. Abbreviations of the samples are the same as in Figure 1.

The specific and overlapping DEGs among the comparisons were visualized in DiVenn (Figure 2). The result showed 463 DEGs were specific to E+NS vs. E−NS, 321 to E+NL vs. E−NL, 961 to E+ES vs. E−ES, and 470 were specific to E+EL vs. E−EL under normal cold condition in the afternoon and evening time. Among the morning time expressed transcripts, 97 DEGs were common between S (E+MS vs. E−MS) and L (E+ML vs. E−ML), of which 42 were upregulated in one but downregulated in other comparison. In addition, there were 556 DEGs were specific to E+MS vs. E−MS and 529 were specific to E+ML vs. E−ML, totaling of 1,085 were significantly up- and downregulated in the morning freezing conditions, and were not expressed in the normal cold condition in the afternoon and evening time (Figure 2).

**Figure 3:**
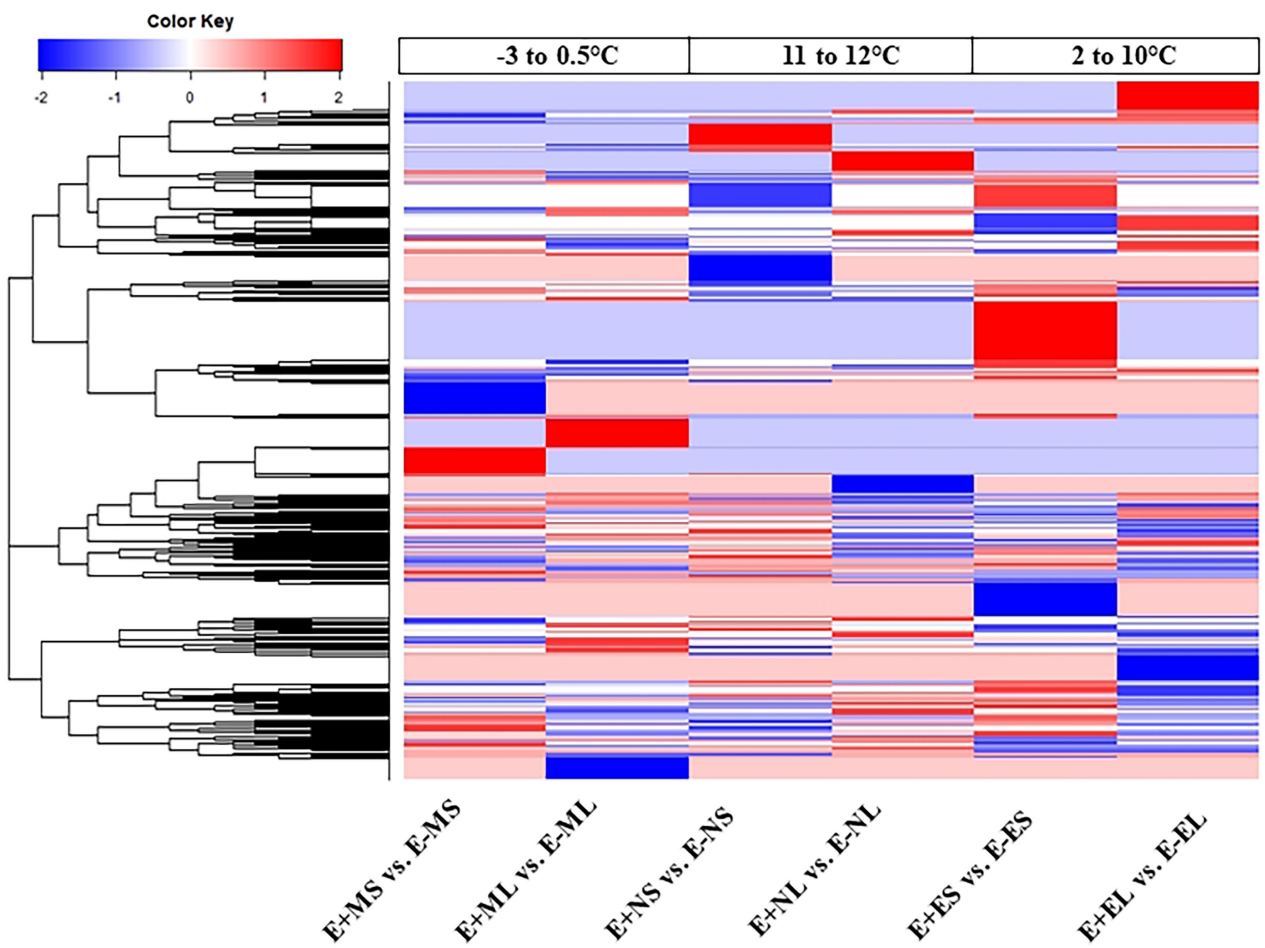
Hierarchical clustering analysis of all differentially expressed genes from six comparisons. Each column represents a different sample with subject to endophyte status, cold stress, and tissue type. Red represents upregulated; blue, downregulated; and white, no change. Abbreviations of the samples are the same as in Figure 1.

The DEGs were used for linkage hierarchical clustering analysis (Figure 3). A distinct pattern of gene expression at transcriptional level under the three time points was observed. Cluster analysis showed that some genes upregulated in the morning were downregulated in the afternoon and evening time or vice versa. Heat map also showed that the expression profiles of the majority genes were different between the S and L tissues in all the time points (Figure 3). This result indicates that tall fescue responded to the stress conditions in time- and tissue-specific manners.

### Gene Ontology Analysis of DEGs

**Figure 4:**
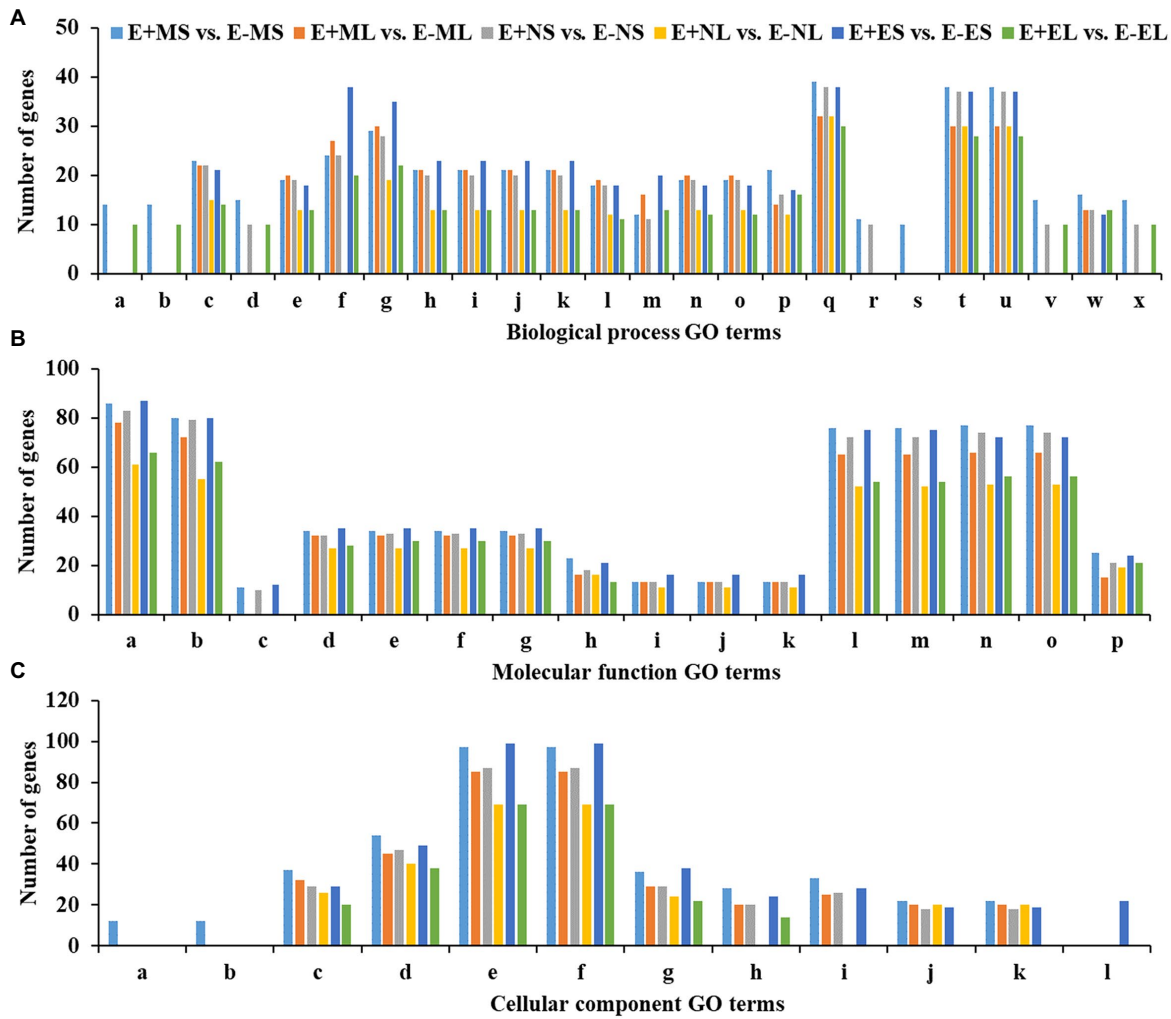
Top 10 significant GO terms in each of the six comparisons obtained using differentially expressed genes of rice orthologues. Significantly (*p* ≤ 0.01) enriched GO terms of **(A)**—biological process (BP), **(B)**—molecular function (MF) and **(C)**—cellular component (CC) are shown in *x*-axis and the number of genes of each GO term are displayed in *y*-axis. The GO terms for the biological process are (a) protein complex biogenesis, (b) protein complex assembly, (c) cellular nitrogen compound metabolic process, (d) macromolecular complex assembly, (e) amine metabolic process, (f) carbohydrate metabolic process, (g) small molecule metabolic process, (h) oxoacid metabolic process, (i) organic acid metabolic process, (j) carboxylic acid metabolic process, (k) cellular ketone metabolic process, (l) cellular amino acid metabolic process, (m) cellular carbohydrate metabolic process, (n) cellular amine metabolic process, (o) cellular amino acid and derivative metabolic process, (p) macromolecule localization, (q) localization, (r) tRNA metabolic process, (s) amino acid activation, (t) transport, (u) establishment of localization, (v) cellular component assembly, (w) protein localization, and (x) macromolecular complex subunit organization. The GO terms for the molecular function are: (a) nucleotide binding, (b) purine nucleotide binding, (c) protein tyrosine kinase activity, (d) nucleoside-triphosphatase activity, (e) pyrophosphatase activity, (f) hydrolase activity, acting on acid anhydrides, in phosphorus-containing anhydrides, (g) hydrolase activity, acting on acid anhydrides, (h) ATPase activity, (i) ATPase activity, coupled to transmembrane movement of substances, (j) ATPase activity, coupled to movement of substances, (k) hydrolase activity, acting on acid anhydrides, catalyzing transmembrane movement of substances, (l) adenyl nucleotide binding, (m) purine nucleoside binding, (n) purine ribonucleotide binding, (o) ribonucleotide binding, and (p) substrate-specific transporter activity. The GO terms for the cellular component are: (a) membrane coat, (b) coated membrane, (c) membrane part, (d) membrane, (e) cell, (f) cell part, (g) cytoplasm, (h) protein complex, (i) macromolecular complex, (j) integral to membrane, (k) intrinsic to membrane, and (l) cytoplasm part. Abbreviations of the samples are the same as in Figure 1.

Out of 5,757 significant DEGs, 1,099 got hit to 732 rice genes in the six comparisons, E+MS vs. E−MS, E+ML vs. E−ML, E+NS vs. E−NS, E+NL vs. E−NL, E+ES vs. E−ES and E+EL vs. E−EL and were used for GO analysis (Supplementary Table 3). We obtained 98 significant GO terms of three major categories, such as BP, MF, and CC (Supplementary Tables 4, 5), and only the top 10 significant GO terms of each of the six comparisons were presented in Figure 4. Some of the identified GO terms were often contained common genes. Though our key objective was to identify DEGs under different time points, we tried to understand the function of genes those were expressed under the freezing temperatures (−3–0.5°C) in the morning *via* GO analysis. In E+MS vs. E−MS samples, 38 GO terms under BP, 31 under MF, and 11 under CC categories were identified. The most significant GO terms involved are “protein complex biogenesis” and “protein complex assembly” under BP; “nucleotide binding” and “purine nucleotide binding” under MF; and “membrane coat” and “coated membrane” under CC (Figure 4; Supplementary Figure 4; Supplementary Table 5). There were 86 genes under the “nucleotide binding” category (Figure 4), of which four genes: *LOC_Os04g58410.1* (tall fescue gene ID: Fa.36660.1), *LOC_Os03g51030.1* (Fa.8356.1), *LOC_Os03g51030.2* (Fa.8921.1 and Fa.10647.1), *LOC_Os03g54084.1* (Fa.6944.1), are involved in GO:0006974 (cellular response to DNA damage stimulus), GO:0033554 (cellular response to stress), GO:0071214 (cellular response to abiotic stimulus), GO:0071478 (cellular response to radiation), and GO:0071482 (cellular response to light stimulus).

Similarly, in E+ML vs. E−ML, GO analysis detected 21 GO terms under BP, 30 under MF, and nine under CC categories. The most significant GO terms involved are “cellular amino acid metabolic process,” “cellular amine metabolic process,” and “cellular nitrogen compound metabolic process” under BP; “pyrophosphatase activity,” “nucleoside-triphosphatase activity,” “hydrolase activity, acting on acid anhydrides, in phosphorus-containing anhydrides,” “hydrolase activity, acting on acid anhydrides,” and “nucleotide binding” under MF; and “membrane part” under CC category (Figure 4; Supplementary Figure 5; Supplementary Table 5). There were 32 genes under each of the GO terms “pyrophosphatase activity,” “nucleoside-triphosphatase activity,” “hydrolase activity,” and “membrane part,” and the other 78 genes under the “nucleotide binding” category (Figure 4), of which three genes, *LOC_Os04g32560.2* (Fa.47164.1), *LOC_Os12g12850.2* (Fa.23585.2) and *LOC_Os02g57530.2* (Fa.32995.1) are involved in GO:0033554 (cellular response to stress), GO:0006974 (response to DNA damage stimulus), GO:0071369 (cellular response to ethylene stimulus) and GO:0071495 (cellular response to endogenous stimulus).

Among the top 10 significant GO terms in each of the six comparisons, there was one significant GO term “amino acid activation” under BP and two GO terms “membrane coat” and “coated membrane” under CC, which were enriched only E+MS vs. E−MS under the freezing stress (−3–0.5°C) in the morning (Figure 4). Most of the genes associated with the three GO categories mentioned above were related to kinase activity, binding, signaling, and transporter activity. Interestingly, there was only one molecular function GO term “lyase activity,” which was not among the top 10 GO terms significantly enriched in E+ML vs. E−ML during the freezing stress in the morning than other cold stresses (Supplementary Table 5). There were 12 genes under “lyase activity” category, of which one gene, *LOC_Os04g37920.1* (Fa.63716.1) was identified in GO:0033554 (cellular response to stress) and GO:0006974 (response to DNA damage stimulus) categories.

### KEGG Pathway Analysis of DEGs

**Figure 5:**
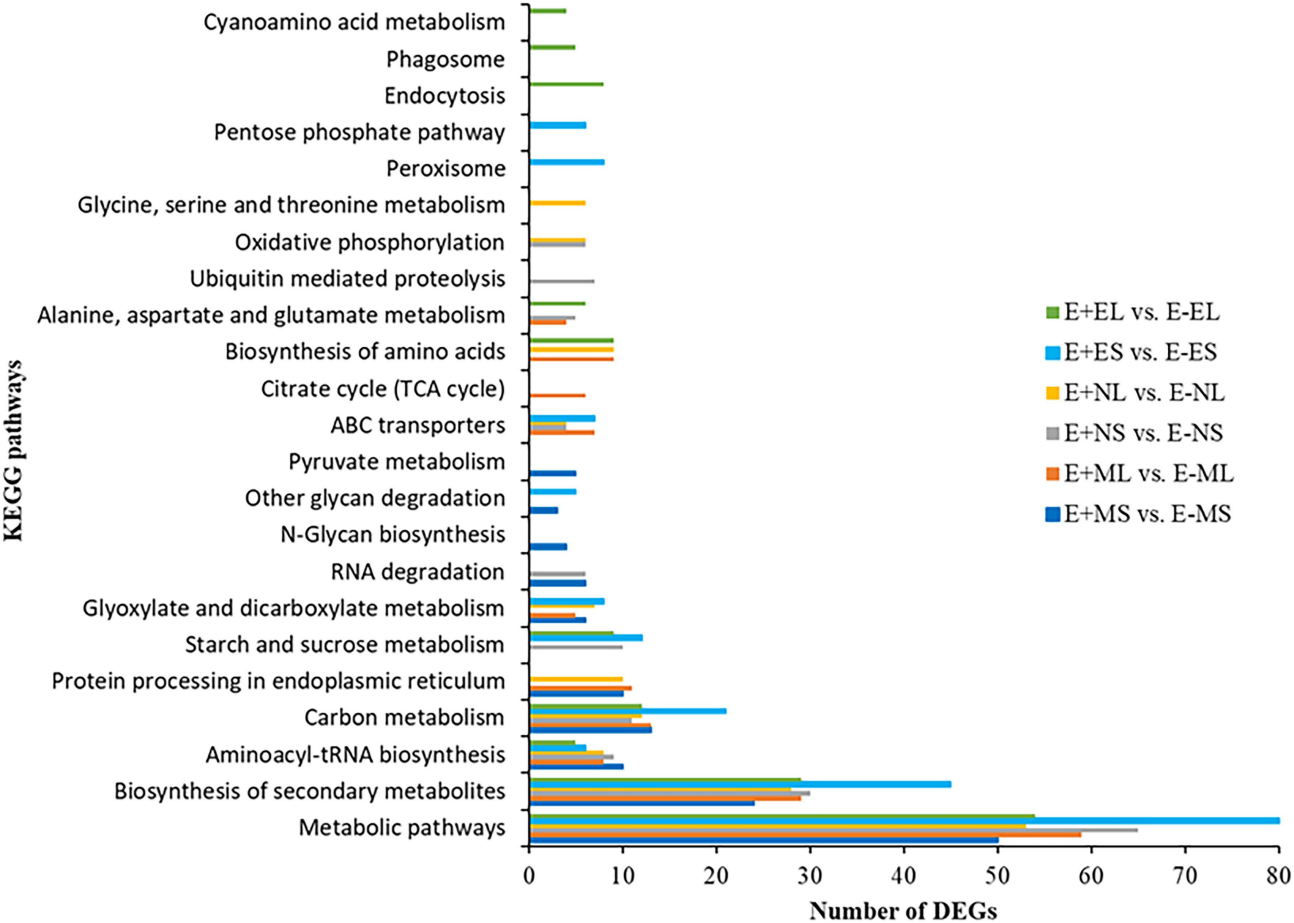
The distribution of differentially expressed genes in KEGG pathways. The top 10 enriched KEGG pathways based on FDR-corrected value of *p* (*p* < 0.05) in each six comparisons, E+MS vs. E−MS, E+ML vs. E−ML, E+NS vs. E−NS, E+NL vs. E−NL, E+ES vs. E−ES, and E+EL vs. E−EL were displayed in *y*-axis and the number of DEGs under each KEGG pathways were displayed in *x*-axis. Abbreviations of the samples are the same as in Figure 1.

We identified 85 and 90 KEGG pathways at E+MS vs. E−MS and E+ML vs. E−ML, respectively under freezing stress in the morning. Similarly, 81 and 71 pathways were identified at E+NS vs. E−NS and E+NL vs. E−NL, respectively under afternoon cold condition, and 86 pathways were identified in both E+ES vs. E−ES and E+EL vs. E−EL under evening cold period (Supplementary Table 6). Top 10 KEGG pathways of each six comparisons were shown in Figure 5. Among the top 10 enriched pathways, “metabolic pathways (dosa01100),” “biosynthesis of secondary metabolites (dosa01110),” and “carbon metabolism (dosa01200)” were contained maximum number of DEGs. Under different time points, metabolic pathway contained lowest number (50) of DEGs at morning freezing stress (E+MS vs. E−MS) and maximum number (80) at evening cold condition (E+ES vs. E−ES). Similarly, biosynthesis of secondary metabolites contained lowest number (24) of DEGs at morning freezing stress (E+MS vs. E−MS) and maximum number (45) at evening cold condition (E+ES vs. E−ES; Figure 5).

### Prediction of Ortholog Genes of Rice as a Potential Candidate Responsible for Cold Tolerance in Tall Fescue

In this study, by annotating the gene function through GO analyses, we identified eight candidate genes specifically expressed in the morning, but neither in the afternoon nor in the evening. Four genes, *LOC_Os03g51030.1* (Fa.8356.1), *LOC_Os03g51030.2* (Fa.8921.1 and Fa.10647.1), *LOC_Os03g54084.1* (Fa.6944.1), and *LOC_Os04g58410.1* (Fa.36660.1) were upregulated (+6 to +22 FC) in S tissues in the morning; and the remaining four genes: *LOC_Os04g37920.1* (Fa.63716.1) and *LOC_Os02g57530.2* (Fa.32995.1) were downregulated (−8 to −27 FC), and *LOC_Os12g12850.2* (Fa.23585.2) and *LOC_Os04g32560.2* (Fa.47164.1) were upregulated (+21 to +38 FC) in L tissues in the morning (Table 2).

**Table 2:** List of eight candidate genes in tall fescue expressed under freezing temperatures in the morning.

Tall fescue gene ID	log2 FC^*^						Important GO terms^ǂ^	Rice orthologs^¥^
	TE + MS vs. TE − MS	TE + ML vs. TE − ML	TE + NS vs. TE − NS	TE + NL vs. TE − NL	TE + ES vs. TE − ES	TE + EL vs. TE − EL		
							Lyase activity	
Fa.63716.1		−26.87					GO:0033554 cellular response to stress GO:0006974 response to DNA damage stimulus	*LOC_Os04g37920.1*
							Nucleotide binding	
Fa.8356.1	+22.11						GO:0071478 cellular response to radiation GO:0071482 cellular response to light stimulus GO:0071214 cellular response to abiotic stimulus	*LOC_Os03g51030.1*
Fa.8921.1	+21.68						GO:0071478 cellular response to radiation GO:0071482 cellular response to light stimulus GO:0071214 cellular response to abiotic stimulus	*LOC_Os03g51030.2*
Fa.10647.1	+22.42							
Fa.6944.1	+7.46						GO:0071478 cellular response to radiation GO:0071482 cellular response to light stimulus GO:0071214 cellular response to abiotic stimulus	*LOC_Os03g54084.1*
Fa.36660.1	+6.56						GO:0006974 response to DNA damage stimulus GO:0033554 cellular response to stress	*LOC_Os04g58410.1*
							Pyrophosphatase activity, nucleoside-triphosphatase activity, hydrolase activity, and nucleotide binding	
Fa.47164.1		+37.57					GO:0006974 response to DNA damage stimulus GO:0033554 cellular response to stress	*LOC_Os04g32560.2*
Fa.23585.2		+20.74		−22.57			GO:0006974 response to DNA damage stimulus GO:0033554 cellular response to stress	*LOC_Os12g12850.2*
							Nucleotide binding and membrane part	
Fa.32995.1		−7.69					GO:0071369 cellular response to ethylene stimulus GO:0071495 cellular response to endogenous stimulus	*LOC_Os02g57530.2*

*The expression values (log2 fold change) in each comparison, important GO terms and rice orthologs were provided*.

* *+, Upregulated; −, Downregulated; FC, Fold change*.

¥ *Rice gene ID was taken from the Rice Genome Annotation Project (rice.uga.edu)*.

ǂ *Hydrolase activity, acting on acid anhydrides, in phosphorus-containing anhydrides; and hydrolase-activity, acting on acid anhydrides referred as hydrolase activity*.

### Validation of DEGs Using qRT-PCR

**Figure 6:**
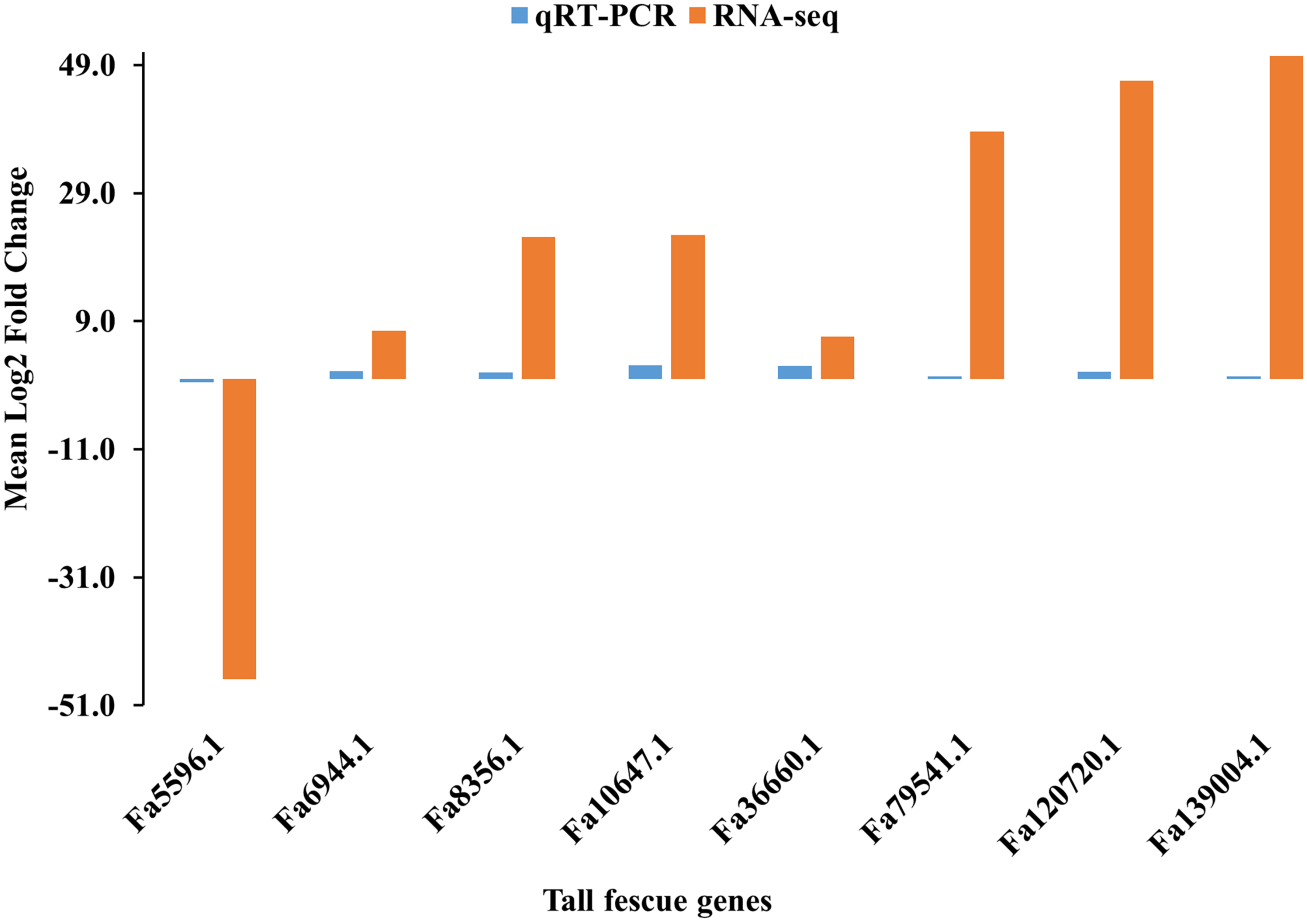
Validation of RNA-seq data obtained from pseudostem of E+ and E− tall fescue collected at morning freezing temperatures through qRT-PCR analysis. The gene expression pattern was shown in *x*-axis and the log2 fold change value was shown in *y*-axis.

To verify the FC values of DEGs identified by pairwise comparison between E+ and E- tissues, eight DEGs, including seven (Fa6944.1, Fa8356.1, Fa10647.1, Fa36660.1, Fa79541.1, Fa120720.1, and Fa139004.1) upregulated and one (Fa5596.1) downregulated in E+MS vs. E−MS were evaluated using qRT-PCR method. The expression levels of the selected DEGs detected with qRT-PCR showed similar pattern with the expression levels calculated from RNA-Seq data (Figure 6), indicating the RNA-seq data were reliable in this study.

## Discussion

The objectives of this study were to characterize the tall fescue transcriptome, and to identify DEGs with and without presence of a fungal symbiont in tall fescue under the cold condition in the natural field environment. Tall fescue is an obligate outcrossing plants such that the established cultivar, Texoma E+ and E- in our experiment is a breeding population with a heterogeneous genetic makeup. Thus, a large sampling size is desirable to accurately capture change in gene expression under the experimental condition. Although the ideal sample size for a large-scale genomic experiment may be debatable, 25–30 individuals per population have been estimated to be adequate for population genetic studies using microsatellite markers (Hale et al., 2012). For each biological replicate in our experiment, we collected tissue samples from 10 individual tall fescue and pooled prior to RNA extraction. Thus, each sample group included 30 plants collectively, which would be sufficient for gene expression profiling. Our study is in agreement with (Schunter et al., 2014), where they suggested that seven individuals can be used for transcriptome sequencing in a non-model fish.

Due to lack of a well-annotated tall fescue reference genome, we generated 36 *de novo* assemblies, consist of S and L tissues of E+ and E- Texoma tall fescue genotypes at three time points and three replications (Table 1). Individual assembly were performed to keep right track of the DEGs in the E+ and E- Texoma genotypes under different cold conditions in the field as well as to know the transcript abundance in the individual samples (Li et al., 2018). Our transcriptome assemblies result in 234,883 transcripts, which may constitute important transcriptomic resources for understanding cold tolerance mechanism of this allohexaploid forage species. In a previous transcriptome study, *de novo* assembly obtained 199,399 contigs from novel endophyte (AR584) infected two tall fescue genotypes under water stress condition in a greenhouse study using the Illumina Genome Analyzer IIx system (Talukder et al., 2015). Recently, Dinkins et al. (2019) generated transcriptome resources from two tall fescue genotypes infected with common toxic endophyte, one with non-toxic strain (NTE19) and the other with hybrid endophyte species (FaTG-4) under water deficit condition in the greenhouse, and assembled against a tall fescue TF153K transcriptome assembly (Dinkins et al., 2017). Both studies are performed in controlled condition in the greenhouse, but we performed this transcriptomic study of AR584 infected Texoma tall fescue at natural field environment. The field condition is always more variable than that of the controlled growth chamber, due to the direct effect of sunlight, day length, soil microbial community, and genotype–environment interaction but can provide more naturalistic outcome. Among the complex interaction in the field, we considered cold stress is the main external environmental factor that contribute to alter gene expression profile in plant when the plants are in stressed.

By comparing the transcript abundance within E+ and E− Texoma tall fescue, we observed that the number of unique transcripts were higher in S than that of L tissue in all three different temperatures (Figure 1). This may be due to the reason that the endophyte colonizes only in the pseudostem but not in leaf blade of continental tall fescue (Takach et al., 2012). More importantly, our results showed that novel endophyte had positive influence on gene expression over E- tall fescue under freezing temperatures (−3.0–0.5°C) in the morning (E+MS: 200,380 > E−MS: 190,514; E+ML: 190,568 > E−ML: 188,760) when the plants were in stress (Table 1). The number of unique transcripts were almost similar in S tissues, but slightly different in L tissues between E+ and E− tall fescue in the afternoon and evening temperature (10–12°C). The higher number of transcripts obtained in E+ over E− samples in all tissues examined under three temperatures conditions in this study (Table 1) might be due to endophyte’s response. Thus, we considered that the plant does not need support from endophyte under normal cold condition for this cool-season grass species, but does need support to perform better under freezing stresses by altering their gene expression. However, previous studies reported that presence of endophyte does not improve the freezing tolerance of perennial ryegrass (Heineck et al., 2018) and the absence of endophyte does not reduce freezing tolerance of tall fescue (Casler and van Santen, 2008) without transcriptome analyses. Similarly, Dinkins et al. (2017) reported that the presence and/or absence of endophyte do not change global expression. Our transcriptome analyses suggest that there is a positive association between freezing tolerance and endophyte infection. This association would provide further insight into the relationship between endophyte infection and its host fitness to abiotic stresses.

DiVenn showed that 1,085 DEGs were specifically expressed only in the morning freezing conditions (Figure 2). Under morning freezing temperatures, plants triggered genes in response to extreme cold stress that was evidenced in GO analyses where three out of top 10 significant GO terms in each of the six comparisons (one under BP and two under CC category) were only found in the morning time (Figure 4; Supplementary Table 5). However, we did not able to analyze all the DEGs due to lack of available information in tall fescue genome and orthologous genes in related species.

The identification of 1,085 DEGs in Texoma tall fescue under morning freezing condition has led us to identify candidate genes responsible for plant survival under cold stress. As tall fescue is a non-model plant species (Talukder et al., 2021), we performed functional annotation of DEGs those got hit to rice ortholog genes through GO analysis and identified eight orthologous genes of rice as candidate genes for cold tolerance in tall fescue. These eight orthologous candidate genes were expressed in the morning freezing temperature only, and are involved in cellular response to stress, cellular response to abiotic, ethylene, and light stimulus and could be a possible route for extreme cold tolerance in tall fescue (Table 2).

Among them, the candidate gene, phytochrome A (*LOC_Os03g51030.1/*Fa.8356.1), phytochrome A (*LOC_Os03g51030.2/*Fa.8921.1 and Fa.10647.1), and phytochrome C (*LOC_Os03g54084.1/*Fa.6944.1) were only upregulated in S under morning freezing temperatures (Table 2; Supplementary Table 3). Light plays an important role in cold acclimation by accelerating the expression of *CORs* in different species and phytochromes have been identified as important factors in the transcriptional control of *CORs* (Lindlöf, 2010). Three types of phytochromes, such as phytochrome A, phytochrome B, and phytochrome C, were identified in the flowering plants (Agrama and Moussa, 1996) that respond to radiation in the environment. In tomato, phytochrome A perceived far-red light to positively and phytochrome B perceived red light to negatively regulate cold tolerance through abscisic acid-dependent jasmonate signaling (Wang et al., 2016). In rice, phytochrome B negatively controls cold tolerance by regulating *OsDREB1* gene expression through phytochrome interacting factor-like protein OsPIL16 (He et al., 2016). The phytochrome C as an essential light receptor for photoperiodic flowering in a temperate grass model *Brachypodium distachyon* (Woods et al., 2014).

The C-terminal small MutS-related (SMR) domain containing protein (*LOC_Os04g58410.1/*Fa.36660.1) identified from the MF GO term “nucleotide binding,” involves in repair of damaged DNA and response to stress, was expressed in S tissue in the morning freezing temperatures (Table 2). The pentatricopeptide repeat (PPR) protein family contain a SMR domain in plants (Liu et al., 2013), and that MutS proteins repair the damaged DNA bases produced during DNA replication processes (Sachadyn, 2010). Recently, Zhang and Lu (2019) reported that the PPR-SMR proteins played an important roles in chloroplast biogenesis and gene expression and retrograde signaling between chloroplast to nucleus. It has been believed that retrograde signaling is necessary when the chloroplast is under stressed (Crawford et al., 2017).

Two other orthologous genes, such as *LOC_Os04g32560.2*/Fa.47164.1 and *LOC_Os12g12850.2/*Fa.23585.2, referred as the ATP-dependent Clp protease ATP-binding subunit clpA homolog CD4B putatively expressed in the chloroplast were upregulated in L tissues under morning freezing temperature. Okuzaki et al. (2021) reported that the acidic domain of the chloroplast ribonucleoprotein 31A (CP31A) is essential for cold tolerance in *Arabidopsis.* Under extreme low temperatures, plant growth and development were ceased and thus plant adjusted to different physiological and biochemical processes in response to cold stress. Exogenous ethylene level altered under cold stress. The putatively expressed ethylene receptor (*LOC_Os02g57530.2/*Fa.32995.1) was downregulated in L tissues under morning freezing temperature. Ethylene levels are negatively correlated with cold tolerance in *Medicago truncatula* (Zhao et al., 2014), but positively affect cold tolerance of tomato (*Lycopersicon esculentum;* Ciardi et al., 1997).

We identified an orthologous gene of rice, FAD binding domain of DNA photolyase domain containing protein (*LOC_Os04g37920.1/*Fa.63716.1), involves in cellular response to stress and repair DNA damage, is similar to cryptochrome (CRY) 1b (Li et al., 2021). CRY receives blue light and causes photomorphogenic responses, for example, cotyledon expansion, prevent hypocotyl elongation, and increased anthocyanin accumulation in plants (Ahmad and Cashmore, 1993). The overexpression of *OsCRY1b* results in short leaf sheath and leaf blade phenotype in rice (Zhang et al., 2006). But, the *CRY1b* (*LOC_Os04g37920.1/*Fa.63716.1) was under expressed (−27 FC) in L tissues under morning temperature in tall fescue. This study would be very useful to develop hypothesis that can bring further understanding of underlying genetics of cold tolerance in tall fescue. By validating the eight DEGs through qRT-PCR analysis, we confirmed that we profiled the expression of DEGs of tall fescue accurately under three different time points in cold condition (Figure 6).

## Conclusion

This study represents the first transcriptome analysis of E+ and E− Texoma tall fescue under freezing and chilling temperatures in the natural field environment. We generated 234,883 unique transcripts from 36 *de novo* assemblies. A total of 5,757 DEGs were identified between E+ and E− samples under three diurnal temperature conditions, of which 1,085 were only up- or downregulated under freezing temperatures in the morning. We were not able to analyze all the genes expressed differentially in two tissues under three temperature conditions, due to lack of available information in related species. Using GO analysis, eight candidate genes were identified from E+ vs. E− samples collected during morning freezing temperature that might help to understand the genetic basis of freezing tolerance in tall fescue. Moreover, the transcriptomic resources generated in this study would serve as valuable resources for grass breeders and to the research community for further structural annotation of tall fescue genome.

## Data Availability Statement

The original research presented in this study is publicly available. The raw RNA-seq datasets can be found online in the National Center for Biotechnology Information (NCBI)’s Sequence Read Archive (SRA) database [https://www.ncbi.nlm.nih.gov/sra] under the accession number: [PRJNA734807].

## Author Contributions

MSI and MS conceived the study. MSI conducted the experiment and wrote the manuscript. TK prepared the library for RNA-seq. MSI and NK analyzed RNA-seq data. MSI and GL prepared RNA for sequencing and performed qRT-PCR. MS critically revised the manuscript. All authors contributed to the article and approved the submitted version.

## Funding

This research work was funded by the Noble Research Institute headquarter in Ardmore, Oklahoma, United States.

## Conflict of Interest

MSI, MS, NK, TK, and GL were employed by Noble Research Institute LLC.

## Publisher’s Note

All claims expressed in this article are solely those of the authors and do not necessarily represent those of their affiliated organizations, or those of the publisher, the editors and the reviewers. Any product that may be evaluated in this article, or claim that may be made by its manufacturer, is not guaranteed or endorsed by the publisher.

## Footnotes

^1^ https://www.pennington.com/

^2^ https://assets.fishersci.com/TFS-Assets/LSG/manuals/1907M_turbodnafree_UG.pdf

^3^ https://www.qiagen.com

^4^ http://csbio-l.csr.uky.edu/ec/

^5^ https://daehwankimlab.github.io/hisat2/

^6^ https://phytozome.jgi.doe.gov/pz/portal.html

^7^ https://www.thermofisher.com

## Supplementary Material

Supplementary Figure 1 Field conditions during sample collection in the morning, afternoon, and evening time. Air temperature data during sampling time was collected from local Mesonet.

Supplementary Figure 2 Experiment design of the transcriptomic study. At each collection time, tissues were collected from three replicates.

Supplementary Figure 3 BLASTx hit of tall fescue transcripts to reference genomes of other plant species. X-axis indicates plant species to identify orthologous genes, and Y-axis indicates number of transcripts/orthologous genes of the respective plant species.

Supplementary Figure 4 Number of significant GO terms under morning freezing stress in E+ versus E− pseudostem tissue. **A**- biological process, **B**- molecular function, and **C**- cellular component GO terms.

Supplementary Figure 5 Number of significant GO terms under morning freezing stress in E+ versus E− leaf blade tissue. **A**- biological process, **B**- molecular function, and **C**- cellular component GO terms.

Supplementary Table 1 List of the primers used in the qRT-PCR experiment.

Supplementary Table 2 List of differentially expressed genes identified in six comparisons, E+MS vs. E-MS, E+ML vs. E-ML, E+NS vs. E-NS, E+NL vs. E-NL, E+ES vs. E-ES and E+EL vs. E-EL. Differentially expressed genes are represented as log2 transformed values. Genes were considered significantly differentially expressed between E+ and E- samples when it passed the significance threshold requiring the adjusted p-value <0.05 and log2 fold change difference ≥ 5. Rice gene ID was taken from the Rice Genome Annotation Project (rice.uga.edu).

Supplementary Table 3 List of rice ortholog genes corresponds to differentially expressed genes 732 identified in six comparisons, E+MS vs. E-MS, E+ML vs. E-ML, E+NS vs. E-NS, E+NL vs. E-NL, E+ES vs. E-ES and E+EL vs. E-EL. Gene name, log2 fold change, rice gene symbol and their description were provided here. Rice gene ID was taken from the Rice Genome Annotation Project (rice.uga.edu).

Supplementary Table 4 Number and identification of significant GO terms (FDR ≤ 0.05) identified in six comparisons using rice orthologues.

Supplementary Table 5 List of significant GO terms (FDR ≤ 0.05) identified in six comparisons using rice orthologs. GO term name, ontology type, description of the GO term, gene number in input list, gene number in background/reference list, p-value, and FDR value of each GO term were provided.

Supplementary Table 6 List of the significant KEGG pathways (FDR corrected p-value <0.05).
