# Supplementary figures and images for: Transcriptome of Endophyte-Positive and Endophyte-Free Tall Fescue Under Field Stresses
Source: Front Plant Sci. 2022 Jun 14;13:803400. doi: 10.3389/fpls.2022.803400 (PMC9237612; doi:10.3389/fpls.2022.803400)

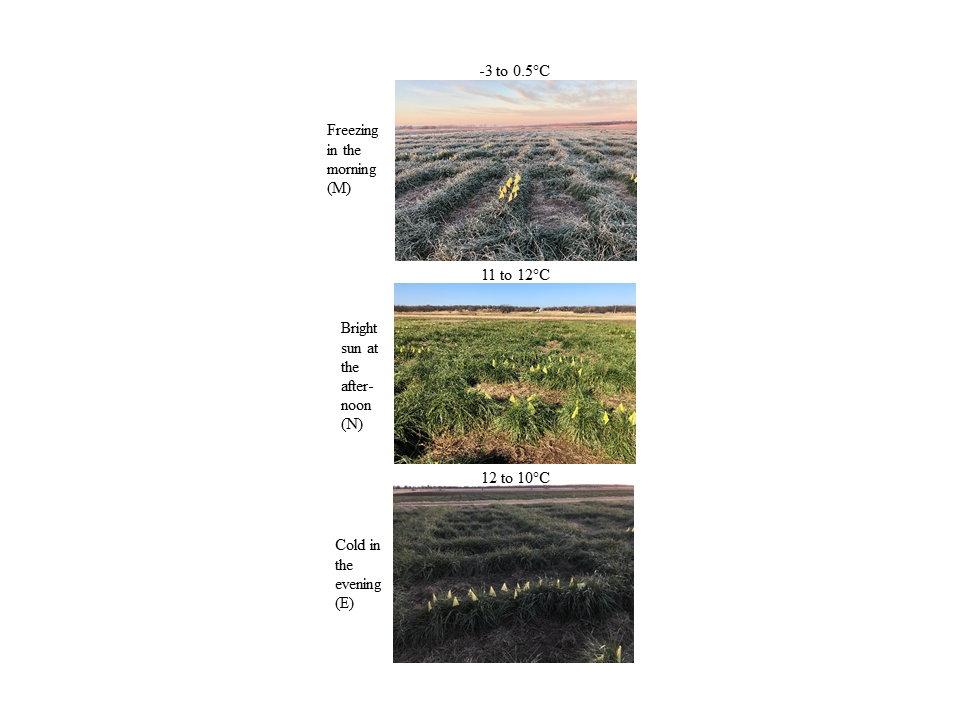

Supplement: Supplementary Figure 1 — Field conditions during sample collection in the morning, afternoon, and evening time. Air temperature data during sampling time was collected from local Mesonet. [file Image_1.TIF]

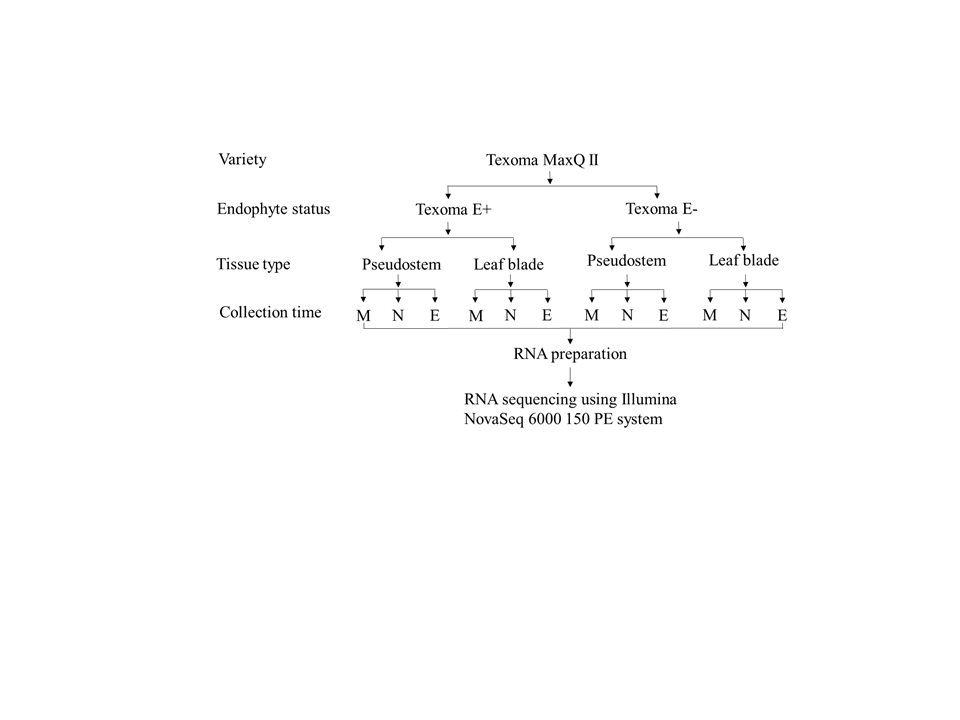

Supplement: Supplementary Figure 2 — Experiment design of the transcriptomic study. At each collection time, tissues were collected from three replicates. [file Image_2.TIF]

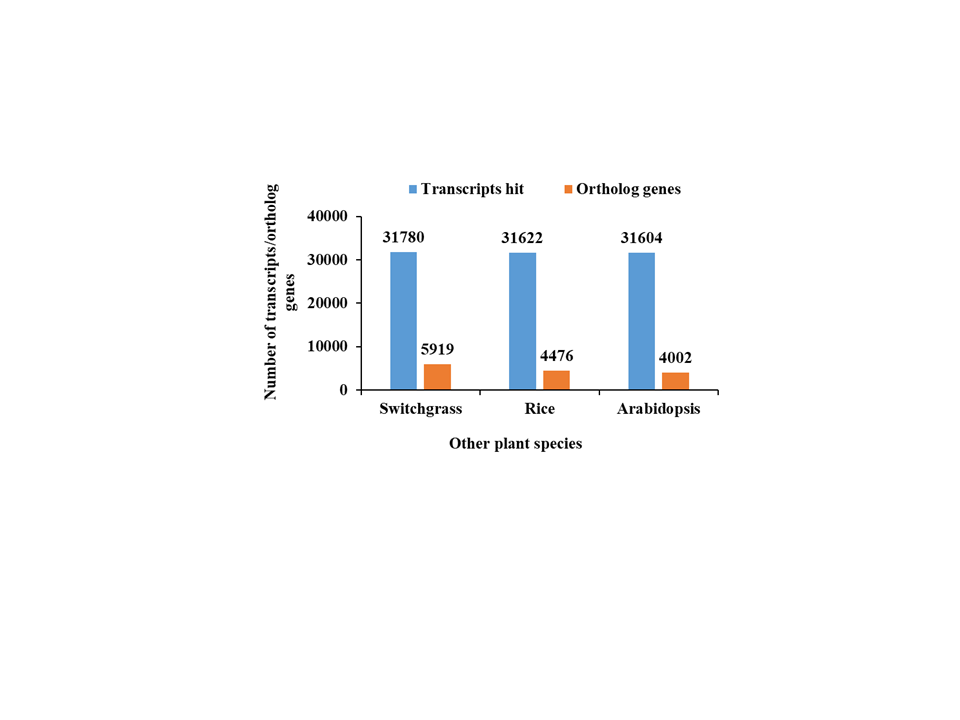

Supplement: Supplementary Figure 3 — BLASTx hit of tall fescue transcripts to reference genomes of other plant species. X-axis indicates plant species to identify orthologous genes, and Y-axis indicates number of transcripts/orthologous genes of the respective plant species. [file Image_3.TIF]

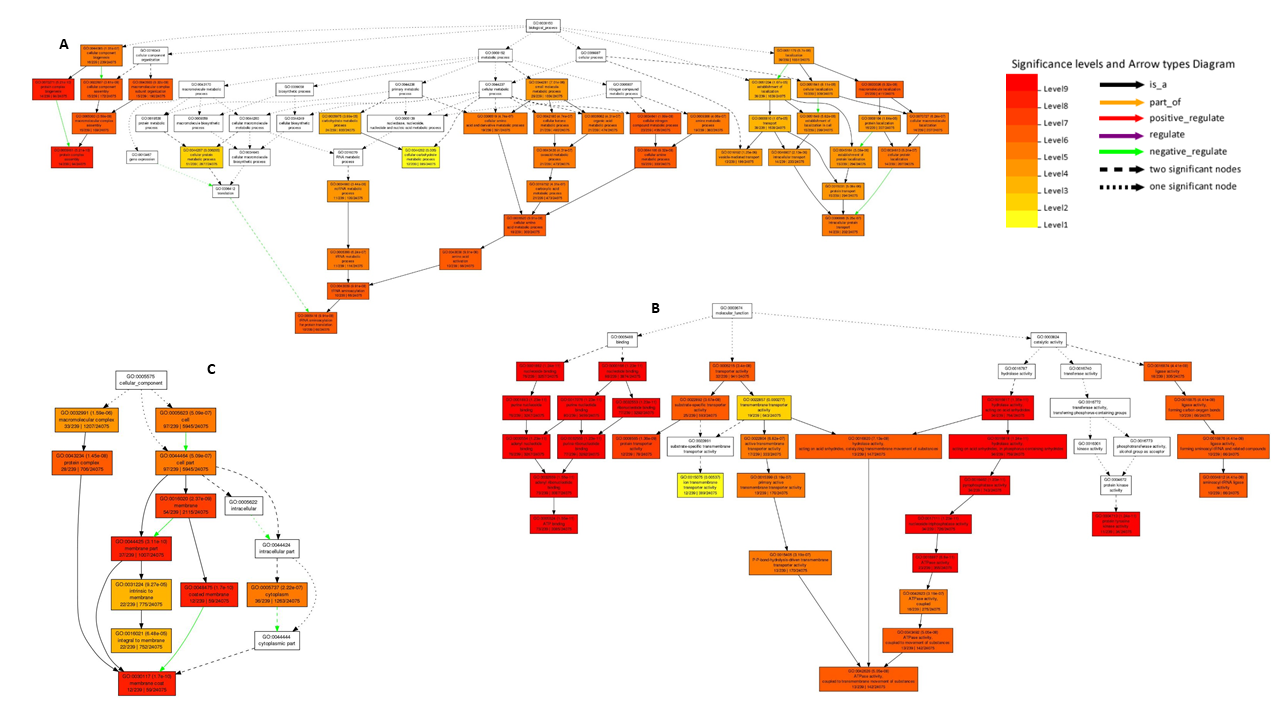

Supplement: Supplementary Figure 4 — Number of significant GO terms under morning freezing stress in E+ versus E− pseudostem tissue. A- biological process, B- molecular function, and C- cellular component GO terms. [file Image_4.TIF]

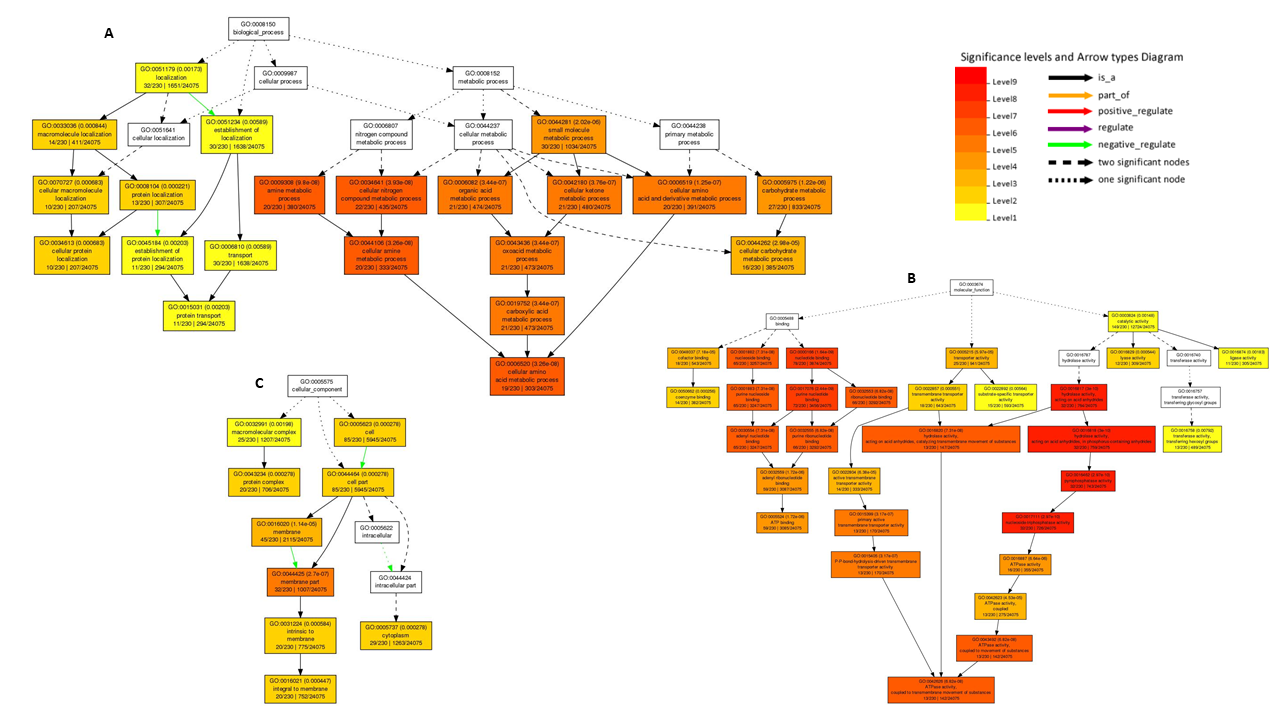

Supplement: Supplementary Figure 5 — Number of significant GO terms under morning freezing stress in E+ versus E− leaf blade tissue. A- biological process, B- molecular function, and C- cellular component GO terms. [file Image_5.TIF]

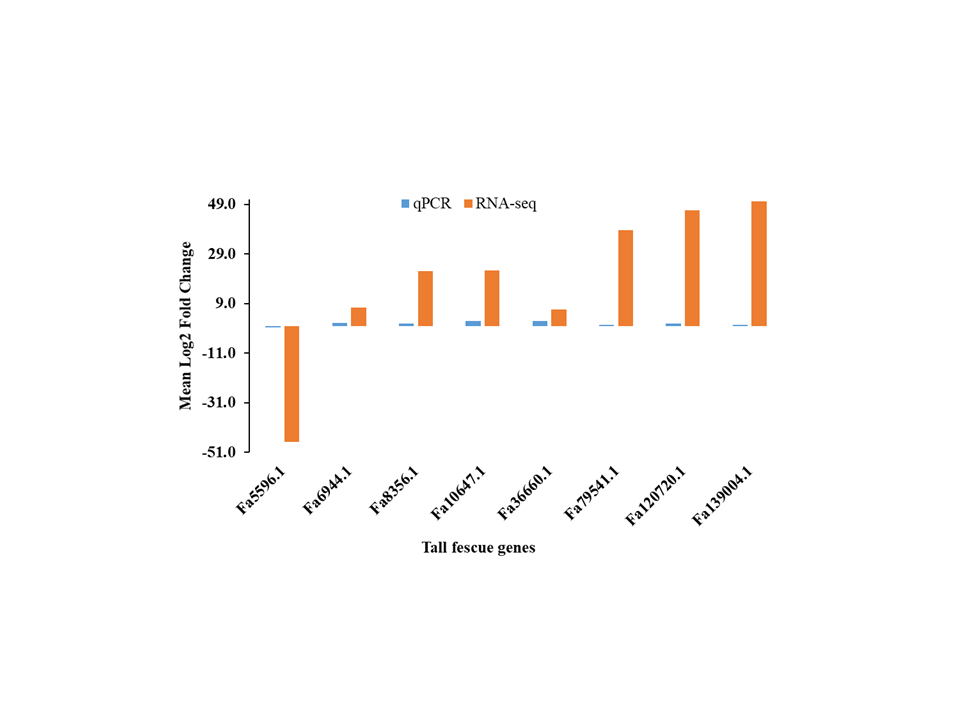

Supplement: Supplementary file 6 [file Image_6.TIF]
