# Supplementary material for: Transcriptome of Endophyte-Positive and Endophyte-Free Tall Fescue Under Field Stresses
Source: Front Plant Sci. 2022 Jun 14;13:803400. doi: 10.3389/fpls.2022.803400 (PMC9237612; doi:10.3389/fpls.2022.803400)
Supplement: Supplementary Table 4 — Number and identification of significant GO terms (FDR ≤ 0.05) identified in six comparisons using rice orthologues. [file Table_4.DOCX]

**Supplementary Table 4** Number and identification of significant GO terms (FDR ≤ 0.05) identified in six comparisons using rice orthologues.

|  | Number of DEGs/rice orthologues | Number of rice orthologues in query list | Significant GO terms^*^ | | | |
| --- | --- | --- | --- | --- | --- | --- |
|  |  |  | BP | MF | CC | Total |
| E+MS versus E-MS | 351/294 | 239 | 38 | 31 | 11 | 80 |
| E+ML versus E-ML | 335/282 | 230 | 21 | 30 | 9 | 60 |
| E+NS versus E-NS | 321/270 | 224 | 33 | 30 | 9 | 72 |
| E+NL versus E-NL | 275/234 | 179 | 16 | 28 | 7 | 51 |
| E+ES versus E-ES | 384/310 | 252 | 29 | 36 | 11 | 76 |
| E+EL versus E-EL | 260/225 | 180 | 27 | 28 | 6 | 61 |
| Non-redundant | 1,099/732 | 588 | 44 | 41 | 13 | 98 |

^*^BP, biological processes; MF, molecular functions; CC, cellular components
